# Changes in Macular Pigment Optical Density after Intravitreal Faricimab in Neovascular Age-Related Macular Degeneration: A Pilot Study

**DOI:** 10.3390/jcm13164893

**Published:** 2024-08-19

**Authors:** Gilda Cennamo, Michele Rinaldi, Flavia Chiosi, Ciro Costagliola

**Affiliations:** 1 Department of Neurosciences, Reproductive Sciences and Dentistry, University of Naples “Federico II”, 80131 Naples, Italy; ciro.costagliola@unina.it; 2 Monaldi Hospital, 80131 Naples, Italy; flaviachiosi@yahoo.it

**Keywords:** intravitreal injections, faricimab, MPOD, SD-OCT

## Abstract

**Background:** This study aimed to evaluate the effects of faricimab intravitreal injections in patients with exudative age macular degeneration (nAMD) after the loading dose using spectral domain optical coherence tomography (SD-OCT) and macular pigment optical density (MPOD). **Methods**: In this observational prospective study, we enlisted a total of 12 consecutive eyes of 12 patients (six females, six males; mean age 70.47 ± 2.46 years) affected by nAMD who consecutively presented to the Eye Clinic of the University of Naples “Federico II” and Monaldi Hospital of Naples, from June 2023 to December 2023. All patients received four once-monthly intravitreal injections of faricimab (6 mg/0.05 mL) (loading phase). At baseline and 1 month after the fourth faricimab monthly injection, all patients underwent assessment of best correct visual acuity (BCVA) and ophthalmic examination, including slit-lamp biomicroscopy, intraocular pressure (IOP), fundus biomicroscopy, SD-OCT, and MPOD. **Results:** A total of 12 eyes of 12 patients (six women, six men; mean age 70.47 ± 2.46 years) were included in this study. A statistically significant raise in BCVA and MOPD parameters was shown between baseline and after the loading phase (*p* < 0.001). **Conclusions**: Intravitreal injections of faricimab led in the short term to a significant functional and MPOD improvement along with a decrease in central macular thickness (CMT) and thus appears to be an effective treatment option without relevant adverse effects. MOPD may be considered as a prognostic factor associated with a good visual prognosis after intravitreal injections treatment.

## 1. Introduction

In developed countries, exudative age-related macular degeneration (nAMD) is a leading cause of irreversible vision loss. Vascular endothelial growth factor (VEGF) antagonists have increasingly become the first-line therapy for nAMD. Anti-VEGF therapy has enabled exudation related to macular neovascularization (MNV) to be resolved [1]. The effectiveness of this treatment had been evaluated by spectral domain optical coherence tomography (SD-OCT), a powerful diagnostic tool for determining retinal thickness and macular thickness. This new bispecific antibody inhibits angiopoietin-2 (Ang-2) as well as vascular endothelial growth factor A [2,3]. The dual neutralization of Ang-2 and VEGF-A channels offers a novel therapeutic strategy for nAMD, which might further normalize the pathological ocular vasculature than anti-VEGF therapy alone [3]. The aim of this prospective study was to determine, using SD-OCT and macular pigment optical density (MPOD), the effects of faricimab intravitreal injections in patients with nAMD after the loading dose, and to assess whether MPOD and its differences might be designed as a possible prognostic factor to predict the visual outcome in these patients who undergo faricimab intravitreal injections. To our knowledge, no studies in the literature have reported the main differences in MPOD after faricimab intravitreal injections, nor have any studies determined whether MPOD is correlated with functional outcomes. This could be of particular interest since, at present, only anatomic findings (such as the recovery of the ellipsoid zone and CMT reduction after intravitreal injections) have been proposed in the literature as possible prognostic factors correlated with the final functional outcome after treatment [4].

## 2. Methods

In this observational prospective study, we enrolled a total of 12 consecutive eyes of 12 patients (6 women, 6 men; mean age 70.47 ± 2.46 years) affected by nAMD who consecutively presented to the Eye Clinic of the University of Naples “Federico II” and Monaldi Hospital of Naples, from June 2023 to December 2023. Criteria for inclusion were age over 50 years and diagnosis of treatment-naïve nAMD due to the presence of type 1, type 2, and type 3 macular neovascularization (MNV). Exclusion criteria included MNV secondary to causes other than nAMD; previous treatments for MNV prior to faricimab (such as laser photocoagulation, photodynamic therapy, intravitreal injections of other anti-VEGF therapy); geographic atrophy; subretinal fibrosis; vitreoretinal disease; retinal vascular disease; myopia over 6 diopters; history of ocular surgery; and significant lens opacity.

Each subject underwent a complete ophthalmological evaluation including assessment of best-corrected visual acuity (BCVA) according to the Early Treatment of Diabetic Retinopathy Study (ETDRS), intraocular pressure (IOP) measurement, slit-lamp biomicroscopy, fundus examination with a +90D lens, fluorescein angiography, indocyanine green angiography, SD-OCT (Heidelberg Engineering, Heidelberg, Germany), and macular pigment analysis (MPOD). The main outcome measures were the different changes in MPOD and CMT after the loading dose of intravitreal injections of faricimab, and relationships between MPOD and functional changes.

All patients received four once-monthly intravitreal injections of faricimab (6 mg/0.05 mL) (loading phase) with a 30-gauge needle. The injection was made through the pars plana under aseptic conditions. At baseline and 1 month after the fourth faricimab monthly injection all patients underwent assessment of BCVA and ophthalmic examination, including slit-lamp biomicroscopy, IOP, fundus biomicroscopy, SD-OCT, and MPOD. Written informed consent for the processing of personal data was obtained from all patients. All procedures performed in studies involving human participants were in accordance with the ethical standards of the institutional research committee and with the 1964 Declaration of Helsinki and its later amendments or comparable ethical standards. Informed consent was obtained from all individual participants included in the study. The research protocol was approved by ClinicalTrials.gov identifier, NCT05969418. 

### 2.1. Macular Pigment Analysis 

For the assessment and analysis of macular pigment, we used the one-wavelength fundus reflectance method to evaluate macular pigment density (Visucam 200; Zeiss Meditec, Jena, Germany). The Visucam is a fundus camera that uses narrow-band wavelength reflectance to measure macular pigment density. Retinal areas containing macular pig-ment absorb more blue light than the rest of the retina. In the blue reflectance image, the degree of darkening is a measure of MPOD. The patients’ pupils were dilated with one drop of 1% tropicamide, and fundus color photographs at 45° were obtained 30 min after pupil dilation. Head alignment was maintained with chin head straps, and an internal fixation target with a central position was used for image alignment. The retina was illuminated with blue light, and only the blue channel of the capture sensor was used for the MPOD image; this suppresses unwanted autofluorescence signals in the green wavelength range. The MPOD was measured in a 30° field of the fundus photograph with the flash level set on automatic and flash intensity set at 12. The MPOD signal was calcu-lated in a range of 4–7° of eccentricity around the fovea, spanning the region where the majority of xanthophylls are concentrated. The optical density and distribution of macular pigment were calculated using a dedicated software algorithm, and the following fundus reflectometry measurements within each image were recorded: mean MPOD and reproducibility, maximum MPOD value, MPOD area (the area within which the macular pigment was detected and defined on the background), and MPOD volume (the sum of all optical densities within the MPOD area). Mean MPOD is the ratio of volume to area and referred to the mean MPOD xanthophylls in relation to the surface area. Maximum MPOD refers to the maximum MPOD xanthophylls (usually in the fovea). MPOD is measured in density units [5,6,7,8,9].

### 2.2. Statistical Analysis 

Statistical analysis was performed with the Statistical Package for Social Sciences (version 20.0 for Windows; SPSS Inc., Chicago, IL, USA). The Mann–Whitney U test was used to evaluate differences in BCVA, central macular thickness (CMT), and MPOD volume, between baseline after four once-monthly intravitreal faricimab injections. The relationships between CMT and BCVA and MPOD parameters were estimated by Pearson’s correlation. 

A *p*-value of <0.001 was considered to be statistically significant.

## 3. Results

A total of 12 eyes of 12 patients affected by nAMD (six women, six men; mean age 70.47 ± 2.46 years) were included in this study. All demographic data are in Table 1. A statistically significant increase in BCVA and MOPD parameters was shown between baseline and after the loading phase (*p* < 0.001). Analysis of the SD-OCT parameters revealed that CMT was significantly reduced after the loading phase in comparison to baseline (*p* < 0.001) (Table 2). We found a significant correlation between mean postoperative MPOD volume and BCVA. In addition, BCVA and MPOD volume at both baseline and after the loading dose did not correlate with the CMT (Table 2).

**Figure 1:**
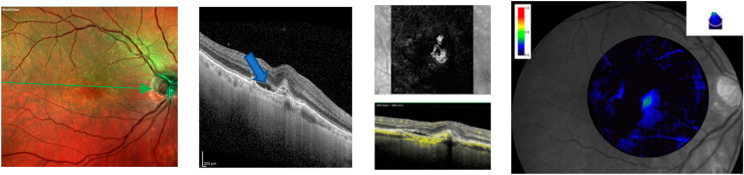
Multicolor imaging, spectral optical coherence tomography (SD-OCT), OCT angiography (OCTA), and macular pigment (MPOD) of active macular neovascularization in the right eye of a male 70-year-old patient before intravitreal injections of faricimab. The blue arrow shows intraretinal fluid and upper reflective material at the level of the external limiting membrane (ELM) and ellipsoid zone (EZ). OCTA reveals a dense vascular network in the macular region. MPOD shows a marked reduction in MPOD volume (blue color).

**Figure 2:**
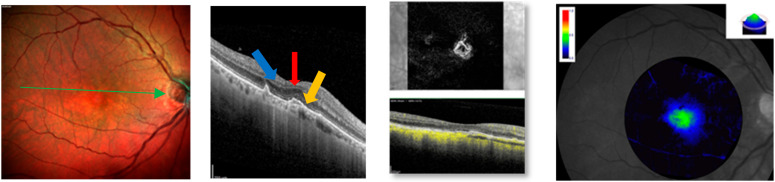
Multicolor imaging, structural SD-OCT, OCTA, and macular pigment of the same eye after loading phase of faricimab. SD- OCT shows a resolution of subretinal fluid (blue arrow) and integrity of ELM (red arrow) and EZ (yellow arrow). OCTA reveals a reduction in the branching capillary network and MPOD shows an increase in macular pigment volume (green color).

A notable improvement in the integrity of the external limiting membrane (ELM) was observed in all of the study participants. SD-OCT imaging revealed a discernible reconstitution of the ELM architecture, characterized by a more distinct and continuous hyper-reflective band in the outer retinal layers (Figure 1 and Figure 2). This phenomenon was observed across loading dose follow-up time points, suggesting a positive and sustained response to faricimab. Concomitant with the improvement in ELM integrity, our results also demonstrated a significant restoration of the ellipsoid zone (EZ) in the treated eyes (Figure 2). The EZ, corresponding to the junction between the inner and outer photoreceptor segments, exhibited a more well-defined and continuous reflectivity pattern on SD-OCT scans post-treatment. This finding was particularly evident in regions previously affected by pathology, indicating a positive impact of intravitreal injections of faricimab therapy on the structural integrity of the photoreceptor layer (Figure 1 and Figure 2).

## 4. Discussion

A quantitative and functional analysis of the macula region in patients with exudative AMD before and after four once-monthly faricimab injections was performed, using SD-OCT and MOPD techniques, for the first in this prospective study. Our results revealed a statistical increase in BCVA and in MPOD volume after the loading dose, and these results did not correlate with the SD-OCT parameters, showing that functional recovery may precede the structural improvement.

We hypothesized that the anti-VEGF treatment could impact retinal and choroidal exudation, decreasing vascular hyperpermeability and resulting in a significant decrement in CMT. Previous studies have shown that VEGF is an important factor in terms of increasing vascular permeability because it remodels the blood–retinal barrier, thereby influencing retinal exudation. In particular, VEGF downregulates the retinal pigment epithelium tight-junction proteins and the tight junctions between retinal capillary endothelial cells, which in turn precedes to a breakdown of the outer and inner blood–retinal barriers, respectively, thereby impacting retinal and choroidal exudation [10]. The anti-VEGF intravitreally injected faricimab is able to penetrate these barriers and act on the choroidal and retinal vascularizations, reducing their vascular permeability. 

On the other hand, Ang-2 operates within the broader angiopoietin–tie system, which includes its counterpart Ang-1 and their shared receptor, Tie-2. Ang-2’s impact on retinal vascular permeability is multifaceted. One of the primary mechanisms through which Ang-2 influences retinal vascular permeability is by destabilizing endothelial cell junctions. This destabilization allows for increased paracellular transport of fluids and solutes across the endothelial barrier. Moreover, Ang-2 can enhance the responsiveness of endothelial cells to other permeability-inducing factors, amplifying the overall effect on vascular leakage [11].

Therapeutic interventions targeting the Ang-2 pathway have the potential to restore vascular stability by reducing vascular leakage, neovascularization, and inflammation, as well as vascular responsiveness, to the effects of VEGF-A. Preclinical studies demonstrated marked reduction in vascular leakage and inflammation versus VEGF-A inhibition alone [12].

The statistical increase in BCVA and MPOD parameters in our study may be explained by the early ELM and ellipsoid zone recovery seen at SD-OCT. The early restoration of a normal ellipsoid zone and ELM may suggest morphological and functional recovery of the photoreceptors determining most of the final functional improvement after treatment. This could be due to the significant reduction in CMT as well as the significant improvement in BCVA in our patients at W 16 and is consistent with previously published experience with treatment with IVF [13].

In our study, intravitreal injections of faricimab showed a good safety profile as we did not observe any adverse events. The low rate of adverse events could be due to the positive anti-inflammatory effect of IVF as an Ang-2 inhibitor. 

However, larger multicenter studies with long follow-up are important to ensure the rate of adverse events.

Moreover, it is essential to recognize and address certain limitations inherent in our study design.

First, the study’s reliance on a relatively small population from only two medical centers may restrict the generalizability of the results. A more diverse sample would enhance the external validity of the findings.

Secondly, enrolling only treatment-naïve patients introduces a potential limitation in terms of generalizing the findings to a broader patient population that may include individuals with prior treatment history. Our perspective is to expand the MPOD analysis to refractory nAMD, since patients with previous treatments might have different disease characteristics or responses to the intervention, and the study’s conclusions may not be applicable to those who have received prior therapies.

## 5. Conclusions

In summary, intravitreal injections of faricimab led in the short term to a significant functional improvement along with a decrease in CMT and thus appears to be an effective treatment option without relevant adverse effects. The raise in mean MPOD after intravitreal injections seems to be connected with a good visual prognosis; MPOD could be an early functional biomarker in patients who undergo intravitreal injection. 

Further studies with a larger study group and a longer follow-up are needed to better explain our findings. However, the long-term results have to be awaited to determine if the visual acuity gain and MPOD benefit will be sustained.

## Figures and Tables

**Table 1:** Demographic and ophthalmologic characteristics in patients with exudative age-related macular degeneration before undergoing the loading phase of intravitreal injections of faricimab.

Parameters	
Eyes (n)	12
Sex (male/female) (n)	6/6
IOP	26 ± 1.6 SD
Axial length	21 ± 1.8 SD

Data are expressed as mean ± SD.

**Table 2:** Differences and correlations in spectral OCT (SD-OCT), visual acuity, and macula pigment measurements (MPOD) after loading dose of faricimab.

Preoperative	Postoperative	r	*p*-Value
BCVA (logMAR)	0.7 (±0.3)	0.34 (±0.2)	0.0006
CMT	403.0 (±78.4)	262.0 (±9.3)	0.0001
MPOD volume (d.u. × pixel)	10 825.6 (±3329.7)	14 569.1 (±4764.6)	0.0001
MPOD-BCVA(logMAR)		–0.348	0.0001
CMT-BCVA(logMAR)		0.32	0.042
MPOD-CMT		0.409	0.082

Data are expressed as mean ± SD; BCVA: best-corrected visual acuity; logMAR: logarithm of the minimum angle of resolution; MPOD volume::macular pigment optical density volume; central macular thickness (CMT); Mann-Whitney U test for independent samples; statistical significance *p*-value < 0.001; Pearson’s correlation coefficient.

## Data Availability

The original contributions presented in the study are included in the article, further inquiries can be directed to the corresponding authors.
